# Supplementary figures and images for: Cardiovascular biomarkers as predictors of adverse outcomes in chronic Chagas cardiomyopathy
Source: PLoS One. 2021 Oct 28;16(10):e0258622. doi: 10.1371/journal.pone.0258622 (PMC8553084; doi:10.1371/journal.pone.0258622)

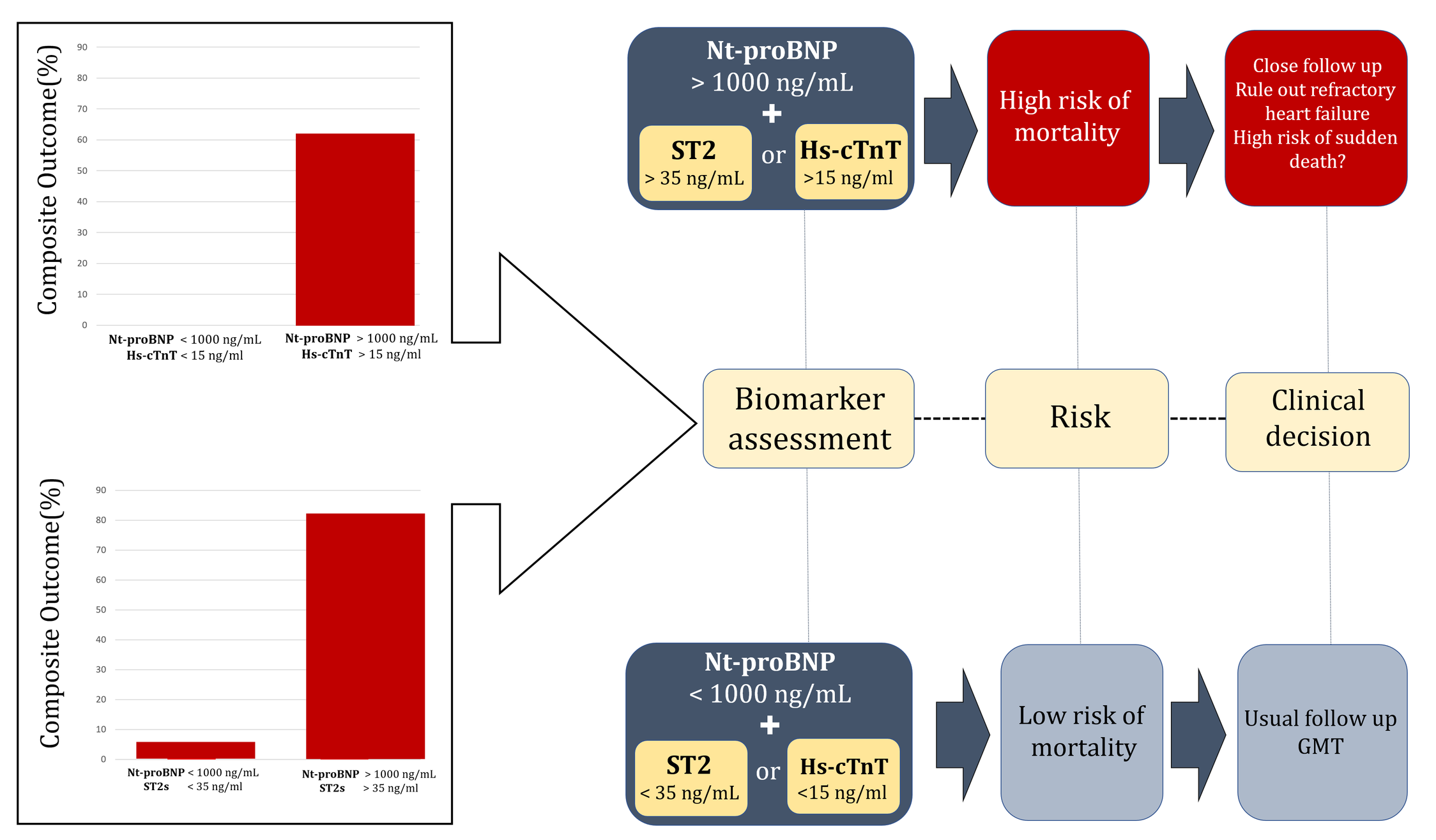

Supplement: S1 Graphical abstract — A multimarker approach combining NT-proBNP and sST2 or hs-cTnT predicted mortality and adverse cardiovascular outcomes accurately after a median follow-up of 52 months in Chronic Chagas Cardiomyopathy. The presence of two of these biomarkers over their cut-off values reflect a higher risk of mortality, indicating the need for a closer follow-up and consideration of advanced therapies. On the other hand, patients with two of these biomarkers under their cut-off values are at lower risk of adverse outcomes, potentially allowing usual follow-up and the maintenance of the guided medical therapy (GMT). (TIF) [file pone.0258622.s001.tif]
