# Supplementary material for: Cardiovascular biomarkers as predictors of adverse outcomes in chronic Chagas cardiomyopathy
Source: PLoS One. 2021 Oct 28;16(10):e0258622. doi: 10.1371/journal.pone.0258622 (PMC8553084; doi:10.1371/journal.pone.0258622)
Supplement: S1 Table — (DOCX) [file pone.0258622.s002.docx]

**Supplemental Material**

**Supplementary Table 1. Prognostic value of the log-transformed biomarker levels in a continuous manner and using cut-off points for the mortality outcome in patients with CCM (n=100)**

| **Biomarker** | **HR*** | **Adjusted Model** | | **AUC** |
| --- | --- | --- | --- | --- |
|  |  | **95% CI** | **p-value** |  |
| NT-proBNP | 1.76 | 1.19 - 2.58 | **0.004** | 83.68 |
| hs-cTnT | 2.22 | 1.38 - 3.56 | **0.001** | 83.92 |
| sST2 | 3.48 | 1.32 - 9.13 | **0.011** | 81.72 |
| Galectin-3 | 1.54 | 0.47 - 5.06 | 0.475 | 82.25 |
| Cys-C | 4.36 | 0.64 - 29.55 | 0.131 | 81.72 |
| NGAL | 1.63 | 0.61 - 4.33 | 0.329 | 81.36 |
| sST2 (>35 vs. ≤35) | 4.04 | 1.55 - 10.47 | **0.004** | 82.49 |
| hs-cTnT (>15 vs. ≤15) | 2.63 | 0.98 - 7.01 | 0.053 | 81.69 |
| Galectin-3 (>16.7 vs. ≤16.7) | 1.18 | 0.44 - 3.18 | 0.734 | 81.42 |
| NT-proBNP (>1000 vs. ≤1000) | 5.12 | 1.02 - 25.79 | **0.047** | 83.09 |
| Cys-C (>1.1 vs. ≤1.1) | 1.07 | 0.33 - 3.46 | 0.914 | 81.30 |
| NGAL (>96.5 vs. ≤96.5) | 1.07 | 0.36 - 3.15 | 0.899 | 81.24 |
| sST2 >35 + NT-proBNP >1000 | 24.21 | 2.35 – 249.29 | **0.007** | 85.88 |
| sST2 >35 + hs-cTnT >15 | 4.25 | 1.61 – 11.24 | **0.004** | 83.32 |
| NT-proBNP >1000 + hs-cTnT >15 | 2.13 | 1.22-3.71 | **0.008** | 85.40 |
| sST2 >35 + NT-proBNP >1000 + hs-cTnT >15 | 23.83 | 2.32 – 244.43 | **0.008** | 85.71 |

*HR adjusted by age and left ventricular ejection fraction.
