# Supplementary material for: Cardiovascular biomarkers as predictors of adverse outcomes in chronic Chagas cardiomyopathy
Source: PLoS One. 2021 Oct 28;16(10):e0258622. doi: 10.1371/journal.pone.0258622 (PMC8553084; doi:10.1371/journal.pone.0258622)
Supplement: Attachment — Submitted filename: Response to reviewers PONE 09062021.docx [file pone.0258622.s003.docx]

RESPONSE TO REVIEWERS

1. Now you stated the refractory heart failure patients were included. Most of them in the group with events. This may explain the high number of events during the first year. This may clearly impact your results and need to be addressed.

**Response:** We understand the reviewer's concerns regarding the high number of events during the first year. Beyond the inclusion of Stage D patients, we must highlight the more severe course of Chronic Chagas Cardiomyopathy compared to other heart failure etiologies. Therefore, a 25% composite outcome incidence in the evaluated period does not necessarily represent an unusual observation in this context. Furthermore, the biomarkers also identified a relevant group of patients with a very low incidence of adverse outcomes. We have now highlighted this relevant aspect in the limitations section.

2. TAble 1 needs to have absolute numbers included with percentagens in brackets to be consistent with the notation presented: N(%) in the first line.

Response: We agree with the reviewer. This has been corrected in the table.

3. An important point that needs clarification is the option for including AGE in the multivariate analysis. It was not significantly disticnt between the two groups, but you selected it. GLS, NYHA class and beta-blockers are highly significant in univariate analysis, but they were not selected. Can you provide an explanation?

Response: We thank the reviewer for this relevant recommendation. We understand his concern, so we evaluated the additive value of including the NYHA class and beta-blockers by comparing the ROC curves of the models. We observed that adding these two variables did not increase the model's ROC area for any of the biomarkers evaluated. Then, we considered that the most parsimonious model, including only age and LVEF, could be optimal due to the small sample size.

4. Use of ICD may have a significant impact on the results. You simply stated that ICD use was not recorded. Since Cardiac transplantations and use of LVAd were described, you certainly had patients with previously implanted ICDs. Sudden cardiac death is the main reason for death in CD, so it is important to clearly identify the causes of death in your sample. An ICD shock is certainly an outcome that will be missed and may affect your results.

Response: We agree with the reviewer on this aspect. However, we were unable to register information regarding ICD shocks. We have now included this in the limitations section.

5. Your figures still need quality improvement.

Response: We agree with the reviewer. This issue has been addressed in the current version. Please download the images directly from the PDF document or the submission system to allow a full-quality view.

6. The results of ROC curves described in methods need to be presented since they are the first step in the selection of those cut-off point used.

Response: We thank the reviewer for his relevant comment. The results of the AUC-ROC for each continuous biomarker assessed can be found in the last column of Table 2.
